# Beyond the calendar: biologic age as a missing vital sign in chronic kidney disease

**DOI:** 10.1093/ckj/sfag140

**Published:** 2026-05-05

**Authors:** Carmine Zoccali, Francesca Mallamaci

**Affiliations:** Renal Research Institute, New York, NY, USA; Institute of Molecular Biology and Genetics, Ariano Irpino, Italy; Associazione Ipertensione Nefrologia Trapianto Renal, c/o Nefrologia, Grande Ospedale Metropolitano, Reggio Calabria, Italy; Associazione Ipertensione Nefrologia Trapianto Renal, c/o Nefrologia, Grande Ospedale Metropolitano, Reggio Calabria, Italy

**Keywords:** biologic age, chronic kidney disease, frailty, risk stratification, transplantation

## Abstract

Chronic kidney disease (CKD) is commonly framed as a model of accelerated aging, yet clinical decisions still rely heavily on chronological age, which poorly captures heterogeneity in functional status, vascular health, cognition, and recovery potential. This narrative review argues that biologic age—a multidomain construct integrating frailty and physical performance, body composition, cardiovascular and vascular markers, inflammatory and metabolic profiles, and cognitive/psychosocial factors—should be treated as a “missing vital sign” in CKD. Such composite measures can refine risk stratification, guide choices between dialysis and conservative care, improve transplant candidate assessment, and inform cardiovascular prevention, ICU triage, and major surgery planning. The article also warns that biologic age metrics must be transparently developed and audited to prevent reinforcing existing inequities and to ensure they trigger supportive, not exclusionary, interventions.

Chronologic age has long served as a convenient shorthand in nephrology. People with chronic kidney disease (CKD) are routinely described as “elderly” or “young,” and dialysis initiation, transplant referral, and even access to intensive cardiovascular prevention are often framed around age thresholds. Yet, people of the same chronological age may differ dramatically in cognitive function, vascular health, metabolism, and physical strength, and clinical systems that treat them as equivalent risk misclassify them, leading to inappropriate care [1]. In CKD, where aging biology is both accelerated and heterogeneous, the gap between chronologic and biologic age is particularly consequential [2, 3].

CKD is frequently portrayed as a model of accelerated aging, marked by an excess burden of vascular calcification, sarcopenia, cognitive impairment, immune dysfunction, and early cardiovascular events even at middle chronologic age [2, 3]. However, every nephrologist knows the contrast between the octogenarian with stage 4 CKD who lives independently and recovers briskly from intercurrent illness, and the patient in their fifties with stage 3 CKD who is frail, functionally dependent, and repeatedly hospitalized. This diversity epitomizes how biologic aging processes, including mitochondrial and immune alterations, vascular stiffening, metabolic change, and epigenetic drift, progress at different rates across people and tissues and are only loosely captured by the calendar [1]. CKD amplifies these processes but does not homogenize them [2, 3].

Nephrologists are already familiar with the limitations of relying on a single surrogate. Estimated glomerular filtration rate (eGFR) is indispensable, yet it is a narrow window on “kidney health”: patients with the same eGFR can differ fundamentally in albuminuria, tubular function, structural damage, and extrarenal complications. Chronologic age operates in a similar way. It is easily ascertained and deeply embedded in risk scores, trial eligibility criteria, and guideline thresholds; however, in practice it serves as a crude proxy for unmeasured variables such as frailty, sarcopenia, vascular and autonomic reserve, cognitive function, and the capacity to recover after stress [1]. Age is often used as a substitute for “functional decline, physiological resilience, accumulated biologic injury, and potential for recovery after illness,” collapsing complex physiology into a single number. In CKD, this double reductionism, with age and eGFR both standing in for multidimensional biology, risks compounding clinical imprecision and widening inequities.

Older adults with CKD are sometimes steered away from kidney transplantation or intensive cardiovascular prevention because of explicit age cutoffs or an impression of “frailty” that has never been formally assessed [4, 5]. At the other end of the spectrum, younger adults with CKD and advanced biologic aging, driven by long-standing diabetes, chronic inflammation, repeated hospitalizations, or cumulative social adversity, may be offered aggressive interventions without adequate attention to limited physiologic reserve, resulting in poor recovery from surgery, recurrent decompensation on dialysis, and heavy treatment burden [6]. Decisions around dialysis versus conservative kidney management sometimes place disproportionate weight on age, rather than on structured assessments of function, symptoms, and goals of care, even though observational data suggest that in very old, highly comorbid, and frail patients, the survival advantage of dialysis over conservative care may be modest and offset by substantial treatment burden [6]. Transplant listing policies may implicitly deprioritize candidates above a given age, despite evidence that pretransplant frailty and functional limitations, not age per se, strongly predict post-transplant mortality and hospitalization [5]. In intensive care unit (ICU) triage for dialysis-requiring acute kidney injury on CKD, older adults may be deemed “too old” despite preserved physiologic reserve, echoing the debates during the Covid-19 pandemic in which geriatricians and ethicists argued that age alone is not an adequate basis for resource allocation [7].

A composite framework that integrates molecular-, immunologic-, metabolic-, and organ-level information to estimate biologic age more accurately than any single metric is needed. Geroscience has begun to furnish such tools. DNA methylation-based “epigenetic clocks” and composite indices such as PhenoAge integrate patterns of methylation associated with morbidity, functional decline, and mortality, moving beyond mere time since birth [2, 8]. In parallel, the concept of “inflammageing” highlights a chronic, low-grade inflammatory and metabolic milieu that links aging, cardiovascular disease, and frailty [3]. These mechanistic insights resonate strongly with CKD, in which chronic inflammation, oxidative stress, immune activation, and vascular injury are central features.

Even without advanced omics, nephrology already has access to pragmatic components of biologic age. Simple frailty and functional measures—gait speed, grip strength, chair-stand performance, and validated frailty indices—predict hospitalization and mortality in dialysis and transplant populations independent of chronologic age [4, 5]. Measures of body composition and muscle strength capture sarcopenia, a core manifestation of accelerated aging in CKD. Cardiac and vascular markers, including arterial stiffness, left-ventricular hypertrophy, natriuretic peptides, and indices of autonomic dysfunction, provide organ-specific information about reserve and vulnerability [7]. Routine inflammatory parameters such as C-reactive protein and interleukin-6, along with biomarkers of oxidative stress and insulin resistance, overlap with the inflammageing profile and are closely linked to adverse outcomes [3]. Cognitive screening and brief assessments of mood, social support, and treatment burden complement these biological measures by capturing the capacity to cope with complex regimens and acute stressors [6]. Taken together, these readily obtainable domains can be assembled into composite scores of physiologic reserve that approximate “biologic age” at the bedside, while more sophisticated biomarkers are refined and validated.

Figure 1 summarizes how such a composite assessment might be deployed at key decision points in CKD. At initial risk stratification and patient counselling, a biologic age-informed profile would help distinguish robust from vulnerable patients at similar eGFR and albuminuria, informing follow-up intensity and preventive focus. When approaching kidney replacement therapy decisions in advanced CKD, the same profile could clarify the likely balance between survival, function, and symptom control on dialysis versus conservative care. In transplantation, integrating standardized assessments of frailty, vascular health, and psychosocial support into candidate evaluation would allow older adults with preserved reserve to be appropriately prioritized, while younger but markedly frail candidates would be channeled toward prehabilitation and more cautious expectations [4, 5]. In cardiovascular prevention, biologic age could help distinguish patients in whom intensive anticoagulation or invasive revascularization is likely to confer net benefit from those in whom competing risks and vulnerability make a conservative strategy more appropriate. Finally, in ICU triage and major surgery planning for patients with CKD, multidomain measures of physiologic reserve can offer a more ethical and accurate basis for decisions than chronologic age alone, aligning with arguments that age should not be the dominant criterion in crisis standards of care [7].

**Figure 1:**
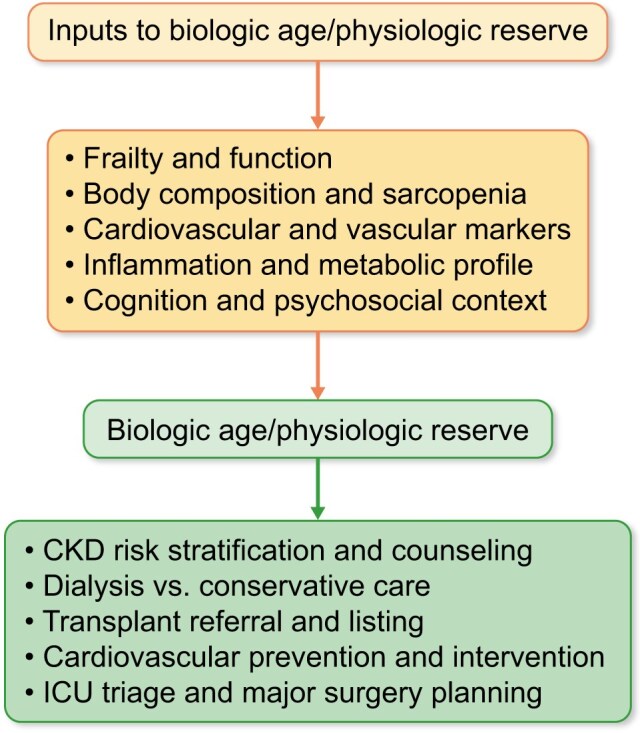
Biologic age assessment at key decision points in CKD. Conceptual framework showing how multidomain inputs inform an integrated estimate of biologic age or physiologic reserve in CKD. Frailty and function, body composition and sarcopenia, CV and vascular markers, inflammation and metabolic profile, and cognition and psychosocial context contribute to a central construct of biologic age/physiologic reserve. This construct then informs clinical decisions about CKD risk stratification and counseling, choice between dialysis and conservative care, transplant referral and listing, CV prevention and intervention, and ICU triage and major surgery planning. CV: cardiovascular.

Any turn toward biologic age must, however, avoid becoming a new instrument of exclusion. In CKD, where inequities in access to nephrology care, transplantation, and dialysis quality are pronounced, biologic age metrics must not become a sophisticated vocabulary for rationing care away from socioeconomically disadvantaged or racially minoritized patients, whose advanced biologic aging often reflects cumulative disadvantage. Instead, high biologic age should trigger targeted interventions: nutritional and exercise programs [9], optimization of anemia and mineral bone disorder, deprescribing of potentially harmful medications, and reinforcement of social support, rather than automatic exclusion from transplantation or intensive therapies. Composite scores should be developed using transparent methods, rigorously validated in CKD cohorts, periodically audited for bias, and tied to clear, nondiscriminatory practice guidance. Incorporating a small set of simple measures—gait speed, grip strength, chair-stand performance, brief cognitive screening, and basic inflammatory markers—into standard CKD follow-up would already represent a significant step toward biologically informed care [3]. Embedding these data in electronic records and using them in pragmatic trials could test whether biologic age-guided strategies improve survival, preserve function, and enhance patient-reported outcomes compared with usual, age-anchored practice. In parallel, CKD cohorts offer an ideal setting to validate cutting-edge aging metrics, including methylation clocks [10] and multi-omic signatures, against outcomes that matter to patients: hospitalization, loss of independence, symptom burden, treatment discontinuation, and the alignment of care with personal goals.

CKD lays bare the limitations of chronological age as a clinical compass. Many patients labeled “too old” for transplantation, intensive cardiovascular prevention, or even dialysis initiation are, in biologic terms, robust and likely to benefit; others, decades younger, are already biologically old. Treating biologic age as a missing vital sign in CKD [8] offers a path to more precise risk stratification, more personalized treatment, and more ethically defensible decisions, aligning nephrology practice with the physiological realities that truly drive outcomes rather than with the relentless but often misleading march of the calendar.
